# Chromosome-level assembly and gene annotation of *Decapterus maruadsi* genome using Nanopore and Hi-C technologies

**DOI:** 10.1038/s41597-024-02912-1

**Published:** 2024-01-13

**Authors:** Longyu Chen, Zhixiong Zhou, Zhiyin Zhou, Junyi Yang, Yacheng Deng, Yulin Bai, Fei Pu, Tao Zhou, Peng Xu

**Affiliations:** 1 State Key Laboratory of Mariculture Breeding, College of Ocean and Earth Sciences, Xiamen University, Xiamen, 361102 China; 2 Fujian Key Laboratory of Genetics and Breeding of Marine Organisms, College of Ocean and Earth Sciences, Xiamen University, Xiamen, 361102 China

**Keywords:** Genome, Genomics

## Abstract

*Decapterus maruadsi* is one of the representative offshore fish in the Western Pacific. Since the last century, it has become a commercially valuable marine fishery species in the Western Pacific region. Despite its high economic value, there is still a lack of high-quality reference genome of *D. maruadsi* in germplasm resource evaluation research. Here we report a chromosome-level reference genome of *D. maruadsi* based on Nanopore sequencing and Hi-C technologies. The whole genome was assembled through 169 contigs with a total length of 723.69 Mb and a contig N50 length of 24.67 Mb. By chromosome scaffolding, 23 chromosomes with a total length of 713.58 Mb were constructed. In addition, a total of 199.49 Mb repetitive elements, 33,515 protein-coding genes, and 6,431 ncRNAs were annotated in the reference genome. This reference genome of *D. maruadsi* will provide a solid theoretical basis not only for the subsequent development of genomic resources of *D. maruadsi* but also for the formulation of policies related to the protection of *D. maruadsi*.

## Background & Summary

*Decapterus maruadsi*, a pelagic fish in the family Carangidae, lives widely in distributed warm offshore waters of East and Southeast Asia^1^. And it is especially abundant along the coasts of the South China Sea^2^. From last century, *D. maruadsi* has become one of the most commercially valuable marine fishery species in Chinese aquaculture. It is also one of the main species captured by pelagic trawls and light-luring fishing vessels^3^. A short lifespan and fast growth and reproduction rates are the most notable features of *D. maruadsi*^4^. Meanwhile, as an r-selection strategy species, it is vulnerable to the environmental deterioration and fishing intensity including those unregulated fishing methods and advanced technologies^5,6^. In recent decades, under the multiple stresses of continuous high-intensity fishing, increasing temperature and feed structure changes caused by global climate change, the population of *D. maruadsi* has been subjected to strong selection pressure, which gradually showing adaptive evolution phenomena such as miniaturization, sex precocity, and the population size has also been decreasing year by year^7,8^. To deal with this dilemma, artificial cultivation of juvenile fish of *D. maruadsi* has been gradually realized in the offshore area of Dongshan island at present.

To ensure the preservation of economically significant species, it is crucial to safeguard their germplasm resources and prevent any potential decline^9^. Genomic data are essential tools for investigating species germplasm resources and assessing population genetic structure and diversity. These resources are of great significance for managing fishery resources and promoting their sustainable use. High-quality reference genomes are essential basic genetic data, and their application value to the aquaculture is also very important. Furthermore, the value of long Nanopore reads which includes low cost, high-throughput sequencing, and high-quality assembly of genomes has been reported by many researches^10,11^. By combining third-generation sequencing and high-through chromosome conformation capture (Hi-C)^12^ technologies, we can assemble the chromosome-level genome rapidly, efficiently and accurately. On this basis, the annotation of *D. maruadsi* genome can be completed. In this report, we provided a high-quality genome assembly of *D. maruadsi* using Illumina short-reads sequencing, Nanopore sequencing, and Hi-C technologies. We obtained a total of 47.61 Gb clean reads by Illumina platform, and through K-mer frequency distribution analysis, the genome size of *D. maruadsi* was about 720.70 Mb. For Nanopore genome sequencing, we assembled a total genome length of 723.69 Mb, which includes a total of 169 contigs. In addition, N50 and N90 lengths of filtered reads were respectively 24.67 Mb and 2.78 Mb, and contigs with a length of 2Kb accounted for 100%. We generated 76.37 Gb of Hi-C filtered data, after chromosome-level scaffolding, there are 23 chromosomes with a total length of 713.58 Mb, resulting in a scaffold N50 of 32.35 Mb. The reference genome of *D. maruadsi* can assist in subsequent population genomics and adaptive genome microevolution studies^13^.

## Methods

### Ethics statement

The *D. maruadsi* in our experiments were collected from Dongshan, Zhangzhou City, Fujian Province, China. Furthermore, the methods used in this work are strictly in accordance with the Guidelines for The Care and Use of Laboratory Animals and followed by the Laboratory Animal Laboratory Committee School of Ocean and Earth Sciences, Xiamen University.

### Sample collection and nucleic acid preparation

A healthy alive female *D. maruadsi* was collected from the Dongshan Pacific Ocean Observation and Experiment Station, Xiamen University. Ten fresh tissue samples, including muscle, eye, skin, gill, kidney, liver, intestine, spleen, heart and stomach, were frozen in liquid nitrogen immediately and then stored in −80 °C. Following the standard protocol of QIAGEN DNeasy Blood & Tissue Kit (Qiagen, Shanghai, China), genomic DNA (gDNA) of muscle was extracted. Total RNA was extracted from ten tissues by a TRIzoL kit (Invitrogen, Shanghai, China) and mixed equally for RNA-seq. The quality of nucleic acid was detected by 1.0% agarose gel electrophoresis and quantified by a Qubit 4.0 fluorometer (Thermo Fisher Scientific, Waltham, MA).

### Library construction and sequencing

For Illumina data, a pair-end sequencing library with 350 bp insert size was constructed using the Illumina TruSeq Nano DNA Library Prep Kit (Illumina, San Diego, CA, USA) and sequenced on the Illumina HiSeq X Ten platform with the 2 × 150 bp read strategy at Novogene company (Tianjin, China). A total of 47.85 Gb raw data were obtained and 47.61 Gb clean data were retained after quality filtering by fastp (V.0.23.1)^14^ software (Table 1). For the Nanopore sequencing, the frozen muscle sample was lysed in SDS digestion buffer, and then the lysate was purified with AMPure XP microbeads (Beckman Coulter, High Wycombe, UK) to obtain High-Molecular-Weight(HMW) gDNA. DNA fragment sizes were selected with the BluePippin system (Sage Science, Beverly, MA, USA) and fragments larger than 20 kb were used for subsequent Nanopore sequencing. The Nanopore libraries were prepared using the Ligation Sequencing Kit (SQK-LSK109, Oxford Nanopore Technologies, Oxford, UK) according to the manufacturer’s instructions and sequenced on the flow cells of the PromethION sequencer at Novogene company (Tianjin, China). Finally, we obtained 86.39 Gb Nanopore data, which average and N50 read length were 22.07Kb and 26.23Kb, respectively. The Nanopore data were further screened before assembly to remove reads less than 1500 bp in length. For Hi-C sequencing, DNA fixed with formaldehyde was digested with the restriction enzyme (*DpnII*), and after being repaired by 5’overhangs biotinylated and blunt-end ligation, these fragments are connected *in situ*, the DNA is cross-linked and purified^15^. In the end, the Hi-C sequencing library was performed on the Illumina HiSeq X Ten platform with a strategy of 2 × 150 bp and generated 76.37 Gb raw reads overall. The RNA-seq library was constructed using Illumina standard protocol (San Diego, CA, USA) and sequenced on the Illumina HiSeq X Ten platform. Finally, we obtained 33.65 Gb paired-end raw reads and 32.61 Gb paired-end clean reads for the following gene prediction (Table 1).

**Table 1:** Statistics for the sequencing data of the *D. maruadsi* genome.

Platform	Insert size (bp)	Raw data (Gb)	Clean data (Gb)	Average Read Length (bp)	N50 Read Length (bp)	Coverage (X)
Illumina	350	47.85	47.61	150	150	66.06
Nanopore	20,000	86.39	—	220,70.2	26,227	119.87
Hi-C	—	76.37	75.77	150	150	105.13
RNA-seq	—	33.65	32.61	150	150	45.25
Total	—	244.26	—	—	—	336.31

Note: Genome size estimated by genome survey (720.70 Mb) were used for sequencing coverage calculation

### De novo genome assembly

The Illumina clean reads were used for further assembly and estimation of genome size using 17-kmer analysis. With K-mer numbers of 434,759,917,857 and a dominant peak depth of 47.24, the genome size was approximately 720.70 Mb, which was similar to the species in Genus *Decapterus* and the heterozygosity and repetitive sequence content were about 0.69 and 32.6%, respectively^16^ (Supplementary Table 1 & Supplementary Fig. 1).

NextDenovo was used for genome assembly based on the overlap layout-consensus algorithm with default parameters. To obtain the contig-level genome, we utilized Racon^17^ for three iterations of polish using the three-generation Nanopore data. Nextpolish^18^ was then employed to correct the genome based on the Illumina data. Lastly, we utilized Purge_Dups^19^ (v.1.25) to de-redundant the genome, resulting in the final contig-level genome. The assembled genome size was 723.69 Mb, including 169 contigs in total, with a contig N50 of 24.67 Mb (Table 2). The assembled genome size almost matched the estimated results of genome survey, which reflected the high assembly integrity.

**Table 2:** Statistics of the *D. maruadsi* genome assembly.

Statistic type	Contig assembly	Chromosome assembly
N50	24,671,527	32,348,610
N90	2,783,646	23,005,116
Contig/Scaffold number(>100 bp)	169	139
Total length	723,689,783	724,050,554
Maximum length	42,935,365	45,095,783
Mean length	4,282,188	31,025,152

**Fig. 1:**
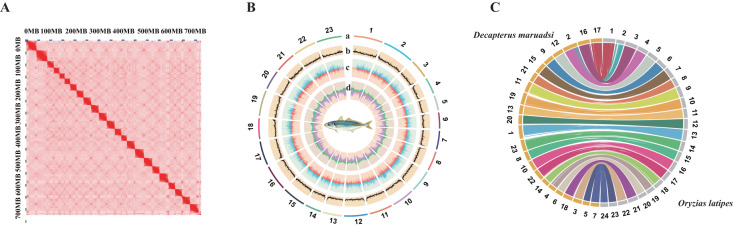
Characteristics of *Decapterus maruadsi* genome assembly. (**A**) Contact map of chromosomal interactions in the *D. maruadsi* genome using Hi-C data. (**B**) A circos plot of 23 chromosomes in *D. maruadsi* genome, the tracks from outside to inside are: a. Lines represent *D. maruadsi* chromosomes; b. GC content; c. Gene density; d. Repeat element density. (**C**) Circos diagram showing synteny relations between *D. maruadsi* and *O. latipes*. Each coloured line represents a 1 Kb fragment match between two species. We reordered the chromosome numbers of *D. maruadsi* for better illustration.

Hi-C sequencing data was used for chromosome assembly of *D. maruadsi*. Firstly, we filtered out Hi-C raw reads(low-quality and duplicated reads) using HiC-Pro^20^. Juicer^21^ was used to map Hi-C clean reads to the reference genome. Subsequently, we used the genomic proximity signal in the Hi-C data sets to get chromosome-level scaffolds. Then, the 3D-DNA pipeline^22^ was used to scaffold the *D. maruadsi* genome. Afterwards, scaffolds were fine-tuned to correct the misassemblies by Juicebox^23^ assembly tools. Finally, we generated a chromosome-level genome assembly of 724.05 Mb and scaffold N50 is up to 32.35 Mb (Table 2). The genome assembly contained 23 chromosomes, with a total length of 713.58 Mb (98.6% of the total length of all contigs). Chromosome sizes ranged from 21.36 to 45.1 Mb, with an average chromosome length of 31.03 Mb (Fig. 1A,B & Table 3).

**Table 3:** Statistics of 23 chromosomes of *D. maruadsi* genome.

Chromosome	Length (bp)	Number of Scaffolds
Chr1	41,317,494	12
Chr2	45,095,783	2
Chr3	26,889,330	5
Chr4	23,735,607	4
Chr5	21,359,908	4
Chr6	22,930,063	4
Chr7	26,730,606	8
Chr8	28,300,000	5
Chr9	31,512,500	4
Chr10	31,747,162	8
Chr11	33,111,663	4
Chr12	33,226,575	9
Chr13	27,185,605	8
Chr14	23,005,116	4
Chr15	33,635,488	5
Chr16	36,885,046	5
Chr17	34,731,214	2
Chr18	29,852,700	6
Chr19	37,058,507	5
Chr20	27,911,733	14
Chr21	29,349,286	6
Chr22	32,348,910	5
Chr23	35,658,500	10
Mean	31,025,152	6
Total	713,578,496	139

### Anotation of repeat sequences

**Fig. 2:**
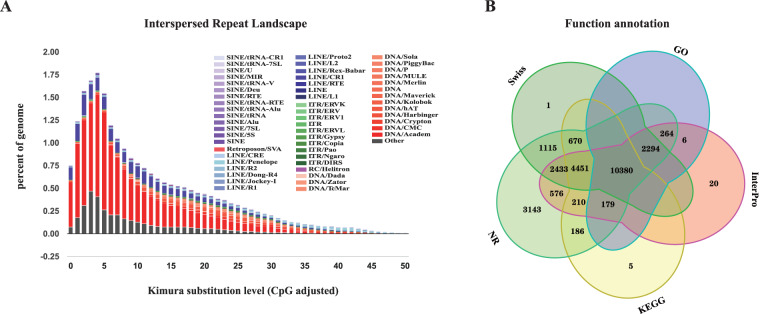
Gene and repeat annotations of the *D. maruadsi* genome. (**A**) Distribution of divergence rate for each type of TEs in the *D. maruadsi* genome. (**B**) Venn diagram of the functionally annotated protein-coding genes based on diferent databases.

Both homology-based and *de novo* methods were used to annotate repeat sequences in the *D. maruadsi* genome. RepeatModeler^24^ (v.2.0.1) and LTR_Finder^25^ (v.1.07) were utilized to detect repetitive sequences in the *D. maruadsi* genome and generate a *de novo* repeat library. Combined with Repbase^26^, the final repeat library was constructed. RepeatMasker^27^ (v.4.1.0) was used to search and classify repeats based on this library. Unclassified repeats were further annotated using TEclass^28^ (v.2.1.3). Transposable Elements (TEs) annotation results were summarized by adopting the buildSummary.pl of RepeatMasker. Moreover, calcDivergenceFromalign.pl was used to calculate the Kimura divergence value of TEs and createRepeatLandscape.pl was used to draw TEs landscapes^29^. To estimate the insertion age, we compared the nucleotide distances between all copies of each TE using the Kimura two-parameter method^29^. We identified tandem repeats using the Tandem Repeats Finder^30^ (v.4.0.9) and soft-masked all repetitive regions except for tandem repeats in the process of protein-coding gene annotation. Finally, a total of 199.49 Mb (27.57% in genome) of consistent and non-redundant repeat sequences were obtained by combining novel, known and tandem repeats. The most abundant repetitive elements were DNA transposons, which spanned more than 102.57 Mb, accounting for 14.17% of the genome of *D. maruadsi*. Besides, the repetitive sequences were also composed of long interspersed elements (LINE) in 37.62 Mb (5.20% in genome), short interspersed nuclear elements (SINEs) in 2.82 Mb (0.39% in genome) and long terminal repeats (LTRs) in 40.99 Mb (5.66% in genome) (Fig. 2A & Table 4).

**Table 4:** Classification of repetitive sequences and ncRNAs of the *D. maruadsi* genome.

Repeat type		Denovo + Repbase Length (bp)	Proportion in Genome (%)
DNA		102,573,344	14.17
LINE		37,618,924	5.2
SINE		2,824,647	0.39
LTR		40,988,688	5.66
Simple Repeat		865,852	0.12
Unkown		398,722	0.06
Total		199,488,111	27.57
**ncRNA type**		**Copy**	**Proportion in Genome (%)**
miRNA		1,285	0.025
tRNA		3,820	0.039
rRNA	18 S	510	0.014
	28 S	150	0.01
	5.8 S	17	0
	5 S	915	0.015
	Total	1,592	0.038
sRNA	CD-box	165	0.003
	HACA-box	85	0.002
	Splicing	501	0.01
	Total	762	0.015

### Prediction and functional annotation of protein-coding genes

For non-coding RNA (ncRNA) annotation, the programs tRNAScan-SE^31^ (v.1.3.1) and RNAmmer^32^ (v.1.2) were used to predict tRNA and rRNA respectively. The other ncRNAs were predicted by searching the Rfam database^33^ (http://eggnogdb.embl.de/). As a result, we annotated four types of non-coding RNAs, including 1,285 miRNAs, 3,820 tRNAs, 1,592 rRNAs and 762 snRNAs (Table 4).

For gene structure prediction, ab-initio strategies, homologous searching and transcriptome-assisted approaches were used to predict protein-coding genes in the *D. maruadsi* genome after soft-masking all repeat sequences. In homology-based prediction, the genetically proximal coding sequences of related species, containing *Oryzias latipes*, *Seriola lalandi*, *Seriola dumerili*, *Oreochromis niloticus*, and *Trachinotus ovatus* were downloaded from European Nucleotide Archive and provided to GenomeTreader^34^ (v.1.7.0) (Supplementary Table 4). Additionally, the RNA-seq data was subjected to the assembly using Trinity^35^ (v.2.10.0). The ab-initio gene prediction was performed using the transcripts assembled from RNA-seq and known genes of *O. latipes*, *S. lalandi*, *S. dumerili*, *O. niloticus*, and *T. ovatus* by Braker2^36^. After two rounds of model training, the optimal parameters are determined. Another gene prediction method involved aligning RNA-seq data to the *D. maruadsi* genome to assemble the transcriptome using Hisat2^37^ and StringTie^38^ (v.2.1.4). Then, the open reading frame (ORF) regions were predicted using TransDecoder (v.5.5.0). Ultimately, EvidenceModeler was utilized to create a thorough gene set, which was then further annotated for protein-coding gene structure via PASA^39^ (v.2.4.1). For functional annotation of predicted gene, Diamond^40^ (v.2.0.6) was applied to align protein-coding genes to the Swiss-Prot (http://www.uniprot.org/), InterPro(https://www.ebi.ac.uk/interpro/) and NR protein databases with E-values < 1*10^−5^. Additionally, GO and KEGG pathway annotations were performed by InterProScan^41^ (v.4.8) (https://www.ebi.ac.uk/interpro/) and KEGG Automatic Annotation Server (KAAS^42^, https://www.genome.jp/tools/kaas/) (Table 5).

**Table 5:** Statistics of gene structure and fuctional annotation of the *D. maruadsi* genome.

Gene structure Annotation	
Number of protein-coding gene	33,515
Average transcript length (bp)	8,571.59
Average exons per gene	7.82
Average exon length (bp)	166.1
Average CDS length (bp)	166.1
Gene fuction Annotation	Number (Percent)
Swissport	21,345(63.69%)
NR	25,902(77.28%)
Interpro	20,813(62.10%)
GO	13,123(39.16%)
KEGG	16,081(47.98%)
Annotated	25,933(77.38%)
Unanotated	7,582(22.62%)

In this study, a high-quality reference genome of *D. maruadsi* was generated, which could provide a solid foundation for species diversity and population genetic studies in the future. Nowadays, genomics is gradually being applied in every stage of large-scale aquaculture production and domestication. As an important aquatic economic fish, *D. maruadsi* is necessary to identify genetic diversity under phenotypic traits by a high-precision chromosome-scale genome to improve the economic benefits of aquaculture species. In addition, high-quality genome-wide maps are important as essential basic genetic data for industrial and scientific research applications, providing a genetic basis and more accurate genetic evaluation tools for the management and sustainable use of *D. maruadsi* fisheries resources. Finally, as a potential cultured fish, the genome of *D. maruadsi* will help to the breeding program for selecting excellent growth-related traits.

## Data Records

The raw sequencing reads of all libraries have been deposited into NCBI SRA database via the accession number of SRP408505^43^. The assembled genome has been deposited at Genbank under the accession number GCA_030347415.2^44^. Moreover, data of the assembled genome and sequence annotations are available at Figshare^45^.

## Technical Validation

### Genome assembly and annotation completeness evaluation

To ensure the accuracy and integrity of the assembly, we assessed the completeness of the final genome assembly using Benchmarking Universal Single-Copy Orthologues (BUSCO)^46^ with the Actinopterygii_odb10 lineage database. Out of 3,640 single-copy orthologues, approximately 97.8% were completely identified in the *D. maruadsi* genome (Supplementary Table 3). Besides, the Illumina short reads were aligned to the genome using the BWA MEM algorithm. Subsequently, employing samtools on the generated BAM files, we calculated the sequencing depth across the genome. The non-zero sequencing depth positions were tallied and summed, then compared to the total base positions for the final coverage percentage. This yielded a mapping ratio of 99.76% and a genome coverage of 98.80% (Supplementary Table 2). Moreover, a total of 33,515 protein-coding genes were successfully obtained by combining ab-initio strategies, homologous searching and transcriptome-assisted approaches. A total of 25,933 genes were successfully functionally annotated in at least one of these databases (Fig. 2B & Table 5). The high integration efficiency, mapping ratio, recognition rate of single-copy orthologues and gene number collectively suggest that the assembled *D. maruadsi* genome was of superior quality.

### Genome assembly accuracy evaluation

To validate the precise arrangement of the *D. maruadsi* genome, we aligned the assembly to the *O. latipes* genome using minimap2^47^ with a unit of 1 Kbp (Fig. 1C). Additionally, we performed the same alignment method with *Trachurus trachurus*, a closely related species in the Carangidae family. The 23 chromosomes identified in the *D. maruadsi* genome showed a significant level of collinearity with the other two species, indicating the high genomic continuity of our assembly (Supplementary Fig. 2 & Supplementary Fig. 3).

Notably, Chromosome 2 of *D. maruadsi* aligns with both chromosome 2 and chromosome 4 of *O. latipes* and *T. trachurus*. To confirm the accuracy of the chromosome number, we performed a nucmer^48^ alignment of chromosome 2 of *D. maruadsi* with chromosome 2 and chromosome 4 of *O. latipes* and *T. trachurus*. The results revealed that chromosomes 4 and 2 of *O. latipes* aligned to the regions 0.34 M - 31.19 M and 31.82 M - 44.96 M, respectively, on chromosome 2 of *D. maruadsi*. Similarly, chromosomes 4 and 2 of *T. trachurus* aligned to the regions 0.09 M - 31.62 M and 31.66 M - 45.06 M, respectively, on chromosome 2 of *D. maruadsi* (Supplementary Fig. 4B). These comparative analyses collectively indicate the presence of a distinct alignment gap within the 31 M - 32 M region of the reported chromosome 2 of *D. maruadsi*. This alignment gap suggests the possibility of a structural alteration and connection region between the two chromosomes in this location.

Moreover, based on the Hi-C assisted assembly data, we have identified that this connection region is completely covered by the precisely assembled contig (Supplementary Fig. 4). Additionally, we selected the genomic range from 31 M to 32 M and utilized the minimap2 tool to align Illumina and Nanopore reads to this region. Subsequently, we implemented a sliding window approach with a window size of 50 kb to calculate the average depth at each genomic position. In this important region, both Illumina and Nanopore data consistently exhibited stable depth profiles, with no significant decrease in depth observed (Supplementary Fig. 5). These observations further emphasize the integrity and continuity of our assembly results.

### Supplementary information

Supplement Figure

Supplement Table

## Data Availability

Genome annotation: (1) RepeatMasker: parameters: -e ncbi -a -nolow -no_is -norna. (2) TE-class: parameters: all parameters were set as default. (3) Braker2: parameters: all parameters were set as default. (4) PASA: --ALIGNERS blat. (5) EvidenceModeler: parameters: all parameters were set as default. Genome assembly: (1) NextDenovo: parameters: all parameters were set as default. Gene family identifcation and phylogenetic analysis: (1) RAxML: parameters: -f a -m PROTGAMMAAUTO. (2) MCMCTREE: parameters: all parameters were set as default. Other analysis modules that were not mentioned parameters were used default parameters. The other custom codes used in this analysis were mentioned in methods sections.
