# Supplementary material for: Chromosome-level assembly and gene annotation of Decapterus maruadsi genome using Nanopore and Hi-C technologies
Source: Sci Data. 2024 Jan 13;11:69. doi: 10.1038/s41597-024-02912-1 (PMC10787795; doi:10.1038/s41597-024-02912-1)
Supplement: Supplementary file 1 — Supplement Figure [file 41597_2024_2912_MOESM1_ESM.pdf]

Contents

Figure S1. Distribution profiles of 17-mer analysis of Illumina reads 2

Figure S2. Dotplot showing synteny relations between *D. maruadsi* and *O. latipes* 3

Figure S3. Dotplot showing synteny relations between *D. maruadsi* and *T. rachurus*...4

Figure S4. *Decapterus maruadsi* chromosome 2 Hi-C contact map zoom-in and species comparison schematic diagram 5

Figure S5. Illumina and Nanopore data depth sliding window on chromosome 2 of *Decapterus maruadsi* 6

Distribution profiles of 17-mer analysis of Illumina reads

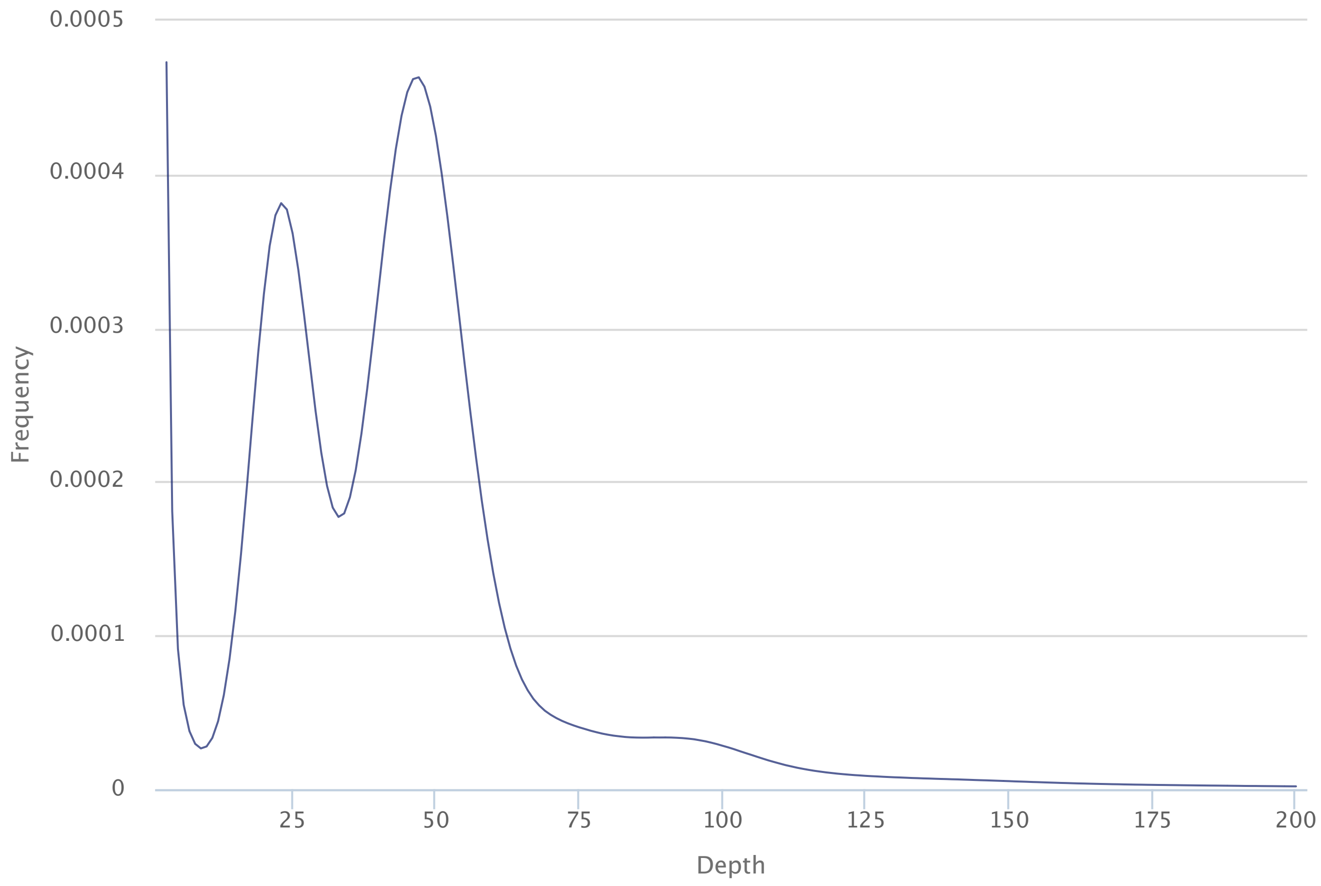

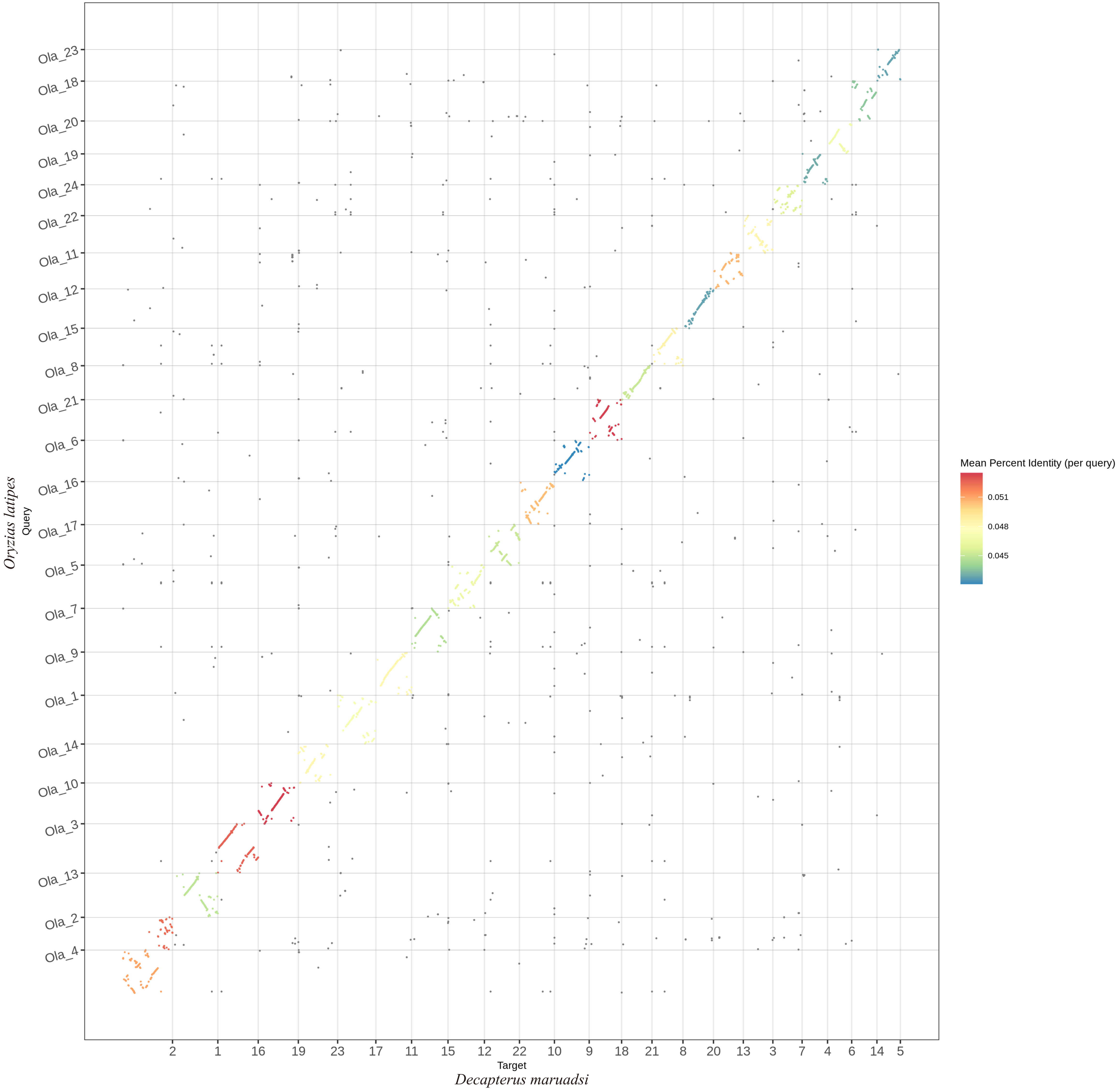

Ola_23
Ola_18
Ola_20
Ola_19
Ola_24
Ola_22
Ola_11
Ola_12
Ola_15
Ola_8
Ola_21
Ola_6
Ola_16
Ola_17
Ola_5
Ola_7
Ola_9
Ola_1
Ola_14
Ola_10
Ola_3
Ola_13
Ola_2
Ola_4
Query
Oryzias latipes
2 1 16 19 23 17 11 15 12 22 10 9 18 21 8 20 13 3 7 4 6 14 5
Target
Decapterus maruadsi
Mean Percent Identity (per query)
0.051
0.048
0.045

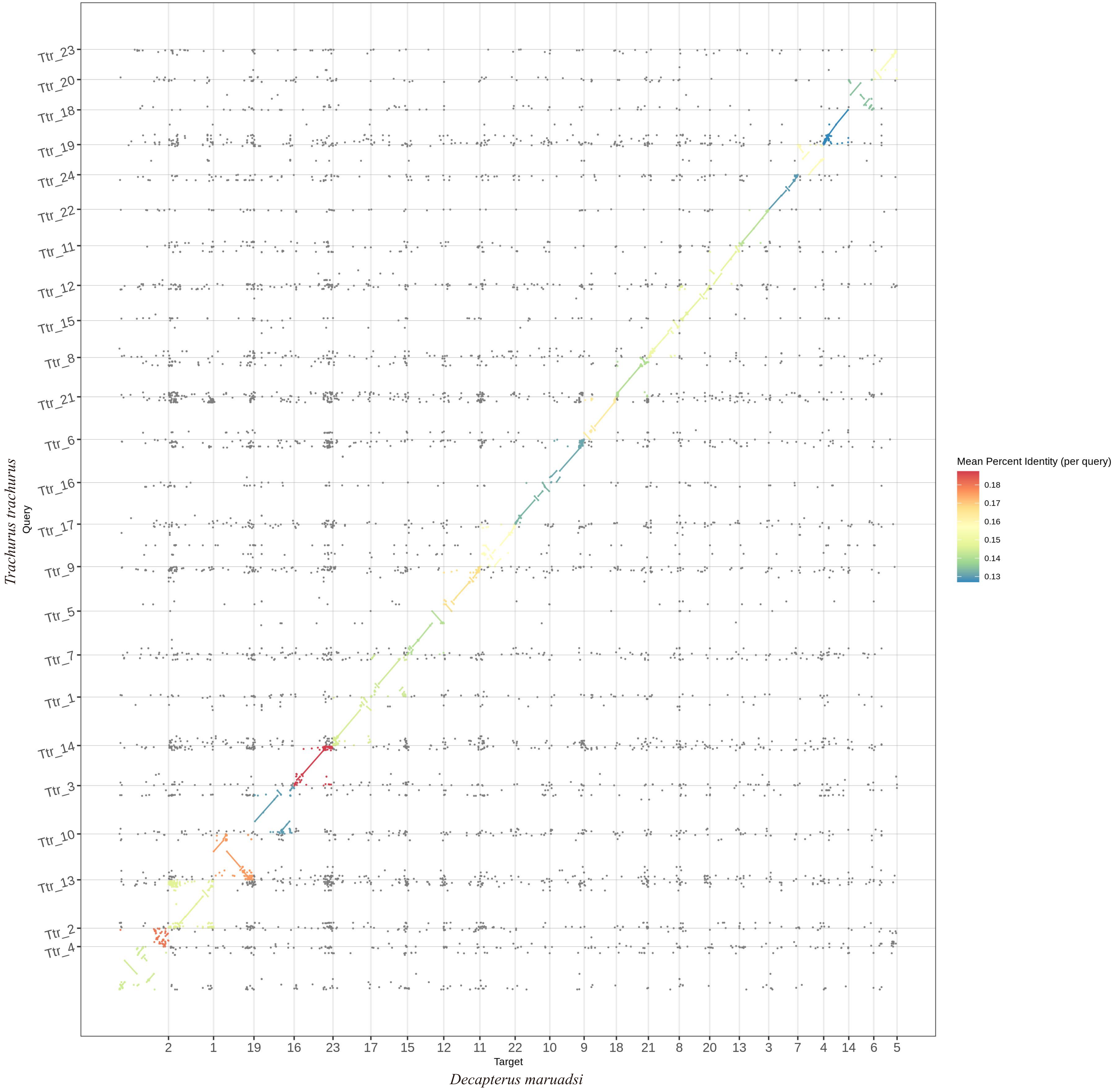

Ttr_23
Ttr_20
Ttr_18
Ttr_19
Ttr_24
Ttr_22
Ttr_11
Ttr_12
Ttr_15
Ttr_8
Ttr_21
Ttr_6
Ttr_16
Ttr_17
Ttr_9
Ttr_5
Ttr_7
Ttr_1
Ttr_14
Ttr_3
Ttr_10
Ttr_13
Ttr_2
Ttr_4
Trachurus trachurus
Query
2 1 19 16 23 17 15 12 11 22 10 9 18 21 8 20 13 3 7 4 14 6 5
Target
Decapterus maruadsi
Mean Percent Identity (per query)
0.18
0.17
0.16
0.15
0.14
0.13

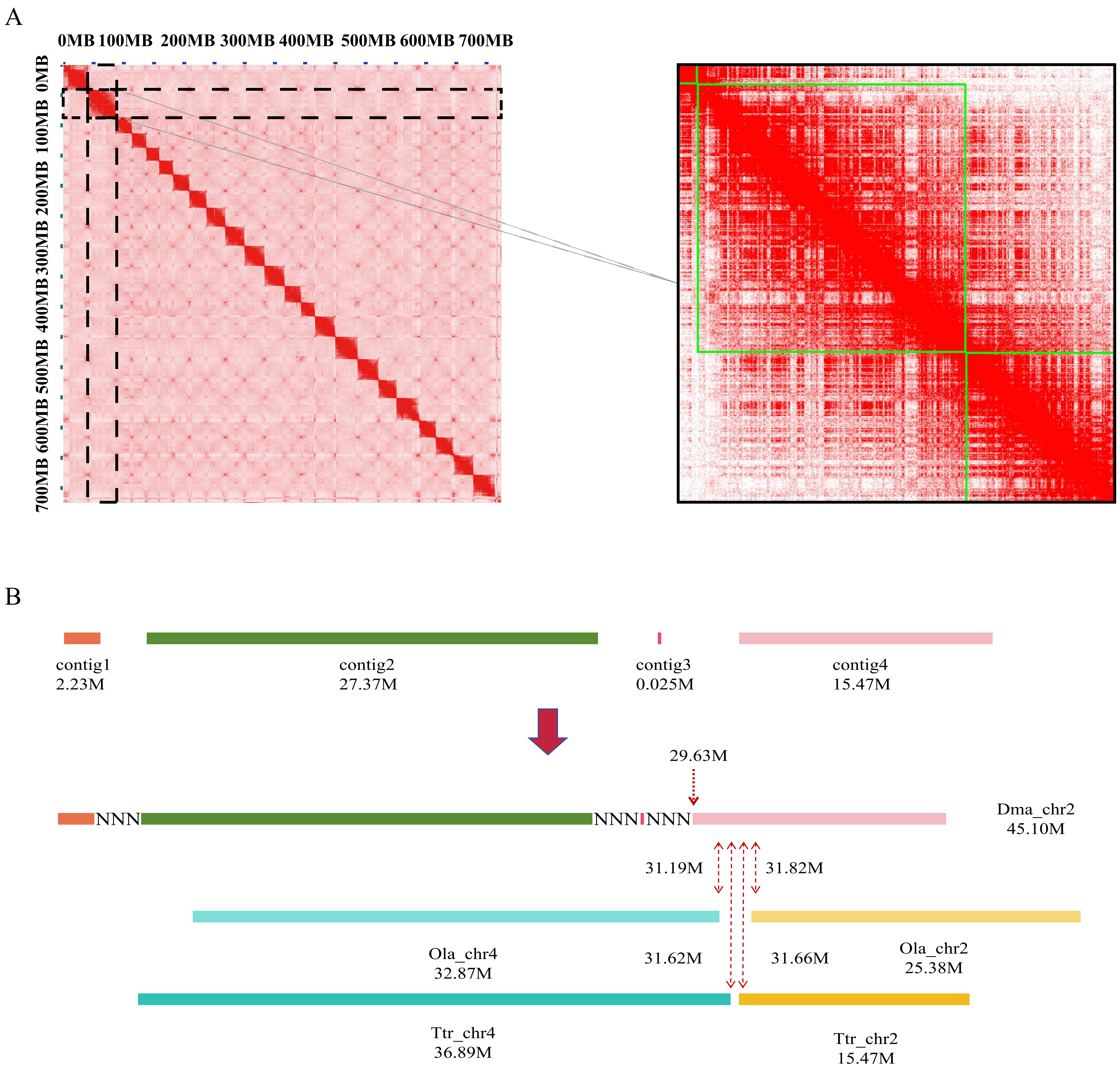
A
0MB 100MB 200MB 300MB 400MB 500MB 600MB 700MB
700MB 600MB 500MB 400MB 300MB 200MB 100MB 0MB
B
contig1
2.23M
contig2
27.37M
contig3
0.025M
contig4
15.47M
29.63M
NNN
NNN
NNN
Dma_chr2
45.10M
31.19M
31.82M
Ola_chr4
32.87M
31.62M
31.66M
Ola_chr2
25.38M
Ttr_chr4
36.89M
Ttr_chr2
15.47M

A

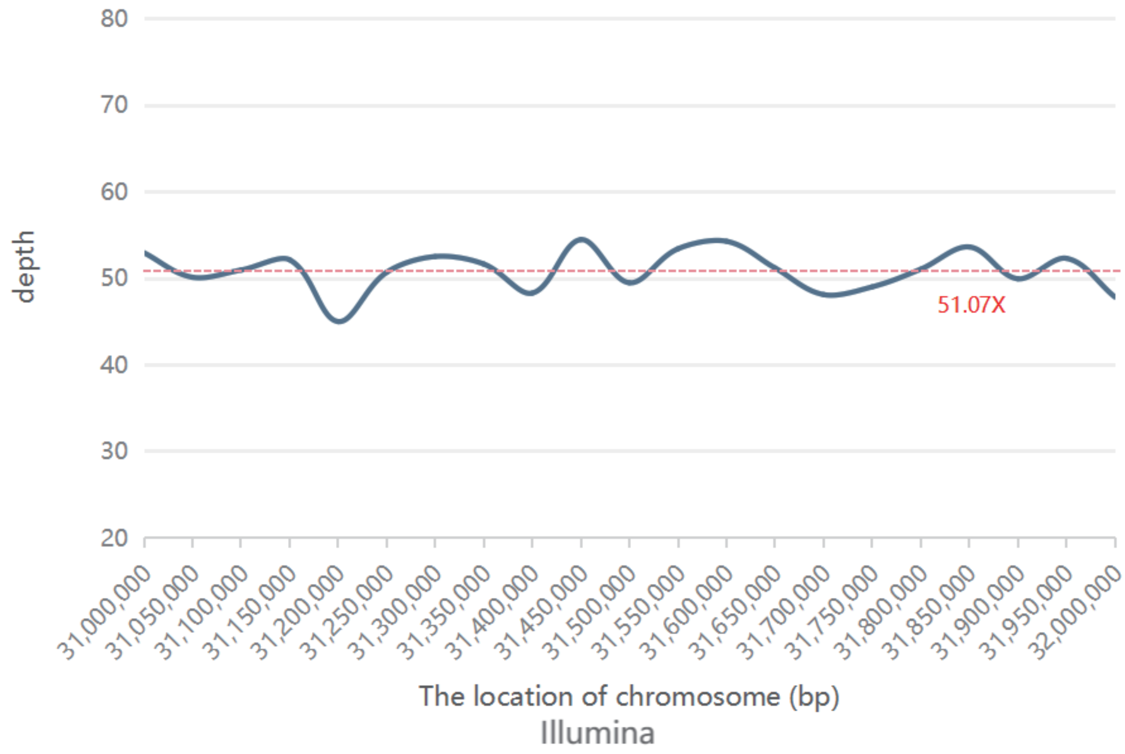

80
70
60
50
40
30
20
depth
51.07X
31,000,000
31,050,000
31,100,000
31,150,000
31,200,000
31,250,000
31,300,000
31,350,000
31,400,000
31,450,000
31,500,000
31,550,000
31,600,000
31,650,000
31,700,000
31,750,000
31,800,000
31,850,000
31,900,000
31,950,000
32,000,000
The location of chromosome (bp)
Illumina

B

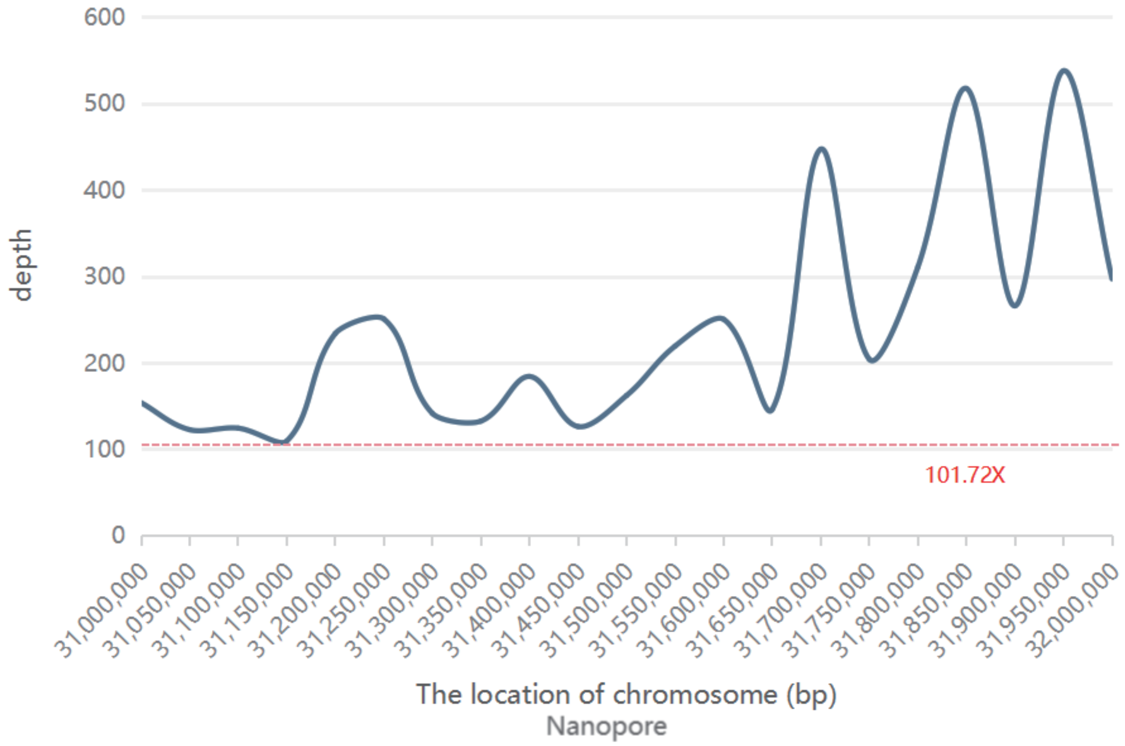

600
500
400
300
200
100
0
depth
101.72X
31,000,000
31,050,000
31,100,000
31,150,000
31,200,000
31,250,000
31,300,000
31,350,000
31,400,000
31,450,000
31,500,000
31,550,000
31,600,000
31,650,000
31,700,000
31,750,000
31,800,000
31,850,000
31,900,000
31,950,000
32,000,000
The location of chromosome (bp)
Nanopore
